# *Candida albicans* Associated with Periodontal Disease Exhibits Different Clusters of Adhesion Gene and Protease Expression

**DOI:** 10.3390/ijms26178721

**Published:** 2025-09-07

**Authors:** Gloria Luz Paniagua-Contreras, Alan Cano-Kobayashi, Ana María Fernández-Presas, Miguel Ruíz-De la Cruz, Héctor Martínez-Gregorio, Felipe Vaca-Paniagua, Eric Monroy-Pérez

**Affiliations:** 1 Facultad de Estudios Superiores Iztacala, Universidad Nacional Autónoma de México, Tlalnepantla 54090, Mexico; yahiko373@gmail.com; 2 Departamento de Microbiología y Parasitología, Facultad de Medicina, Universidad Nacional Autónoma de México, Ciudad de México 04510, Mexico; presas@unam.mx; 3 Unidad de Biomedicina, Facultad de Estudios Superiores Iztacala, Universidad Nacional Autónoma de México, Tlalnepantla 54090, Mexico; miguel.ruiz@iztacala.unam.mx (M.R.-D.l.C.); hector.martinez@iztacala.unam.mx (H.M.-G.); felipe.vaca@iztacala.unam.mx (F.V.-P.); 4 Laboratorio Nacional en Salud, Diagnóstico Molecular y Efecto Ambiental en Enfermedades Crónico-Degenerativas, Facultad de Estudios Superiores Iztacala, Universidad Nacional Autónoma de México, Tlalnepantla 54090, Mexico

**Keywords:** periodontal disease, *ALS*, *SAP*, virulome expression

## Abstract

*C. albicans* has recently been described as a secondary colonizer associated with periodontal infections. This study aimed to determine the expression patterns of *ALS* and *SAP* family genes in *C. albicans* strains isolated from patients with periodontal disease (n = 268), and a control group of healthy individuals without any clinical signs of periodontal disease (n = 100) was included. *C. albicans* and the *ALS* and *SAP* genes were identified using polymerase chain reaction (PCR). An in vitro infection model was used with the strains using the human gingival fibroblast cell line. RNA was extracted using a QIAcube robotic workstation (Qiagen). A QuantiTect Reverse Transcription Kit (Qiagen) was used for first-strand *cDNA* synthesis. *ALS* and *SAP* gene expression in the strains was determined using real-time PCR. A total of 82.5% (n = 66) of the *C. albicans* strains were isolated from patients with moderate periodontitis, 10% (n = 8) from patients with chronic periodontitis, and 7.5% (n = 6) from patients with gingivitis. In the group of healthy individuals, *C. albicans* was identified in 9% (9/100). Overall, the most frequently expressed *ALS* genes in the strains from the three diagnoses were *ALS1* (77/80), *ALS3* (67/80), *ALS4* (67/80), *ALS6* (77/80), *ALS7* (62/80), and *ALS9* (73/80), while the most frequently expressed *SAP* genes were *SAP1* (76/80), *SAP6* (57/80), *SAP9* (78/80), and *SAP10* (77/80). The overall frequencies of expression of the *ALS4*, *ALS9*, *SAP2*, *SAP3*, *SAP6*, and *SAP* genes in the strains were statistically different across the three diagnoses. We identified different profiles of expression of the *ALS* and *SAP* genes in the strains of *C. albicans* that contribute directly to the degree of periodontal disease. Therefore, our findings may contribute to improving our knowledge of the molecular mechanisms of *C. albicans* in the pathogenesis of periodontal disease.

## 1. Introduction

*Candida albicans* is an opportunistic fungal pathogen associated with a wide range and various types of infections, such as oral, vaginal, skin, and systemic candidiasis [1], as well as other skin infections and infections associated with medical devices such as catheters [2,3], primarily in immunocompromised patients [4]. *C. albicans* has recently been described as a secondary colonizer associated with periodontal infections [5,6].

Periodontitis is a chronic inflammatory disease primarily caused by the accumulation of bacterial plaques around the tooth surface. In chronic conditions, this leads to the formation of periodontal pockets, destruction of the supporting tissues of the teeth and alveolar bone, and consequent tooth loss [7]. In the United States of America (USA), periodontitis affects more than 40% of adults, and worldwide, the incidence is 11% [8].

Numerous anaerobic bacteria are responsible for periodontal disease, including *Prevotella intermedia*, *Porphyromonas gingivalis*, *Treponema denticola*, *Aggregatibacter actinomycetemcomitans*, *Tannerella forsythia*, and *Fusobacterium nucleatum* [9,10,11]. However, enterobacteria, such as *Enterobacter cloacae*, *Klebsiella oxytoca, Klebsiella pneumoniae* [12], and *Escherichia coli* [13], and the fungus *C. albicans* [14] have also been described as secondary pathogens associated with periodontitis.

The pathogenicity of *C. albicans* is largely mediated by a repertoire of virulence genes that favor the acuteness and/or chronicity of infections. These genes include the *HWP1* gene (hyphal wall protein 1) [15], adhesion genes of the *ALS* family (agglutinin-like sequence) [16] that favor adhesion to host epithelial cells and biofilm formation, *SAP* family genes (secreted aspartyl protease) that increase tissue degradation and evasion of the host’s immune response [17,18,19], the *LIP* gene family (lipases) [20] that participates in the digestion of lipids from the cell membranes for nutrient acquisition, and *PHL* genes (phospholipases) that promote phospholipid degradation in the membrane of host epithelial cells, favoring invasion [21].

The analysis and expression of the different molecular markers of virulence of *C. albicans* causing vaginal and oral infections have been characterized in our previous study [22,23] and by other authors [24,25]. However, information on the role of *C. albicans* as a secondary pathogen associated with periodontal disease is limited.

Therefore, the aim of this study was to analyze the expression patterns of adhesion- and protease-encoding genes in *C. albicans* isolates from patients with gingivitis, moderate periodontitis, and chronic periodontitis. To achieve this, we used an in vitro infection model using a human gingival fibroblast (HGF) cell line, enabling the characterization of the different molecular arrangements of expression of the genotype encoding for adhesins and proteases in *C. albicans* strains relevant to periodontal disease pathogenesis.

## 2. Results

### 2.1. Origin of the Strains

*C. albicans* was identified in 24.6% (n = 80) of the 268 patients with periodontal disease, and other Gram-negative species, such as *Escherichia coli* (n = 50) and *Klebsiella* spp. (n = 13), were also identified as well as Gram-positive bacteria, such as *Staphylococcus aureus* (50/80). A total of 82.5% (n = 66) of *C. albicans* strains were isolated from patients with moderate periodontitis, 10% (n = 8) were isolated from patients with chronic periodontitis, and 7.5% (n = 6) were isolated from patients with gingivitis (Table 1). The association of *C. albicans/E. coli* (n = 3) and *C. albicans/S. aureus* (n = 2) was identified in patients with moderate periodontitis, and *C. albicans/Klebsiella* spp. (n = 1) was detected in a patient with chronic periodontitis. In the group of healthy individuals, *C. albicans* was identified in 9% (9/100), and no other species of enterobacteria or associated Gram-positive bacteria were detected.

### 2.2. Frequency of ALS Genes in the Strains

Generally, the distribution of the presence of *ALS* genes in the strains was independent of the degree of periodontal disease (Table 1). *ALS1*, *ALS4*, *ALS6*, *ALS7*, and *ALS9* genes were identified in 100% (6/6) of the strains from patients with gingivitis, whereas in strains from patients with moderate periodontitis, the most frequent genes were *ALS4* (65/66), *ALS6*, and *ALS7* (64/66) in each case and *ALS9* (63/66). On the other hand, *ALS1*, *ALS4*, and *ALS7* were detected in 100% (8/8) of the strains associated with chronic periodontitis. In the strains from the group of healthy individuals (n = 9), the *ALS1*, *ALS3*, *ALS4*, and *ALS9* genes were detected in 88.8% (8/9), *ALS2* and *ALS5* were detected in 33.3% (3/9), and *ALS6* and *ALS7* were detected in 77.7% (7/9).

### 2.3. Distribution of SAP Genes in the Strains

Ten genes of the *SAP* family (*SAP1–SAP10*; Table 2) were detected in 100% of the strains isolated from patients with gingivitis (6/6), whereas only seven *SAP* genes (*SAP1*, *SAP4*, *SAP6*, *SAP7*, *SAP8*, *SAP9*, and *SAP10*) were identified in 100% (8/8) of the strains isolated from patients with chronic periodontitis. In contrast, only four *SAP* genes (*SAP1*, *SAP6*, *SAP9*, and *SAP10*) were found in 100% (66/66) of the strains recovered from moderate periodontitis. In the strains from the group of healthy individuals, the genes *SAP1*, *SAP4*, *SAP6*, *SAP8*, *SAP9*, and *SAP10* were detected in 100% (9/9); *SAP2*, *SAP3*, and *SAP7* were detected in 88.8% (8/9); and *SAP5* was detected in 77.7% (7/9). The distribution of the presence of *SAP* genes in the strains was independent of the degree of periodontal disease (Table 2).

### 2.4. ALS Gene Expression in the Strains

To simulate *C. albicans* adhesion during periodontal disease, an in vitro infection model was used in the human gingival fibroblast (HGF) cell line, which was infected with *C. albicans* strains. Subsequently, the levels of *mRNA* expressed by the *ALS* genes in the strains of *C. albicans* were studied after the reverse transcription of *cDNA* by real-time PCR.

The *C. albicans* strains that adhere to the HGF cell line present a high expression of *ALS* genes (Table 3). Overall, the genes *ALS3*, *ALS4*, *ALS6*, and *ALS9* are expressed more frequently in the strains of gingivitis and moderate periodontitis in relation to the strains of chronic periodontitis.

*ALS6* and *ALS9* were expressed in 100% (6/6) of the strains isolated from patients with gingivitis, while *ALS1* (5/6), *ALS3* (5/6), *ALS4* (5/6), and *ALS7* (4/6) were highly expressed.

Regarding the strains recovered from patients with moderate periodontitis, the most frequently expressed genes were *ALS1* (64/66), *ALS3* (58/66), *ALS4* (58/66), *ALS6* (64/66), *ALS7* (52/66), and *ALS9* (63/66).

On the other hand, in the strains recovered from patients with chronic periodontitis, the most frequently expressed genes were *ALS1* (8/100), *ALS6* (7/8), and *ALS7* (6/8). A significant association was observed between the expression frequencies of the *ALS3*, *ALS4*, and *ALS9* genes of the strains in relation to the degree of periodontal disease (Table 3).

### 2.5. SAP Gene Expression in the Strains

To simulate cellular damage by *C. albicans* during periodontal disease, after infection of the human gingival fibroblast (HGF) cell line, the levels of *mRNA* expressed by the *SAP* genes in the strains of *C. albicans* were studied after the reverse transcription of *cDNA* by PCR in real time.

The most frequently expressed *SAP* family genes in the strains from patients with gingivitis were *SAP1* (6/6), *SAP9* (5/6), and *SAP10* (6/6; Table 4).

On the other hand, the most frequently expressed genes in the strains from patients with moderate periodontitis were *SAP9* (65/66), *SAP10* (63/66), *SAP1* (62/66), *SAP6* (51/66), and *SAP2* (49/66).

Of the strains isolated from patients with chronic periodontitis, 100% (8/8) expressed the *SAP1*, *SAP9*, and *SAP10* genes. A significant association was found between the expression frequencies of the *SAP2*, *SAP3*, *SAP6*, and *SAP7* genes in the strains of moderate periodontitis in relation to the strains of gingivitis and chronic periodontitis (Table 4).

### 2.6. Unsupervised Hierarchical Clustering

**Figure 1:**
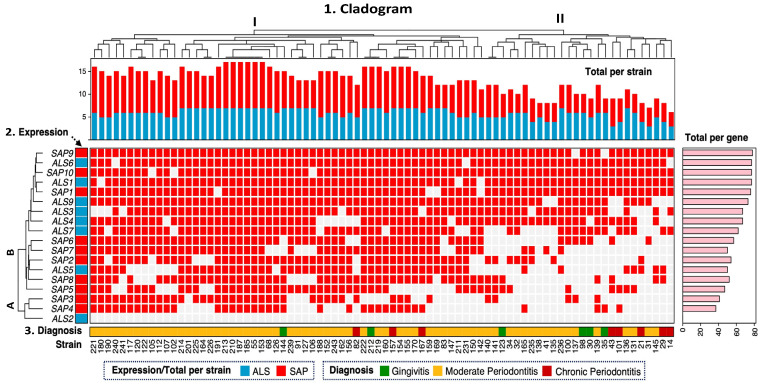
Hierarchical clustering of *C. albicans* strains. The heat map is segmented into three panels. Top panel: (1) Overall cladogram of the distribution of virulence genotype expression across group I and II strains. Middle panel: (2) *ALS* and *SAP* gene expression across strains. A and B expression groups of the *ALS* and *SAP* genes in the strains. Bottom panel: (3) Diagnosis and origin of strains. The right panel (pink) shows the absolute frequency of expression by virulence genotype (*ALS* and *SAP*). Gene expression across strains is represented in red, and absence is represented in white.

Unsupervised hierarchical clustering analysis showed a wide distribution and diversity in the expression of adhesion (*ALS*) and protease (*SAP*) markers in *C. albicans* strains. The overall cladogram of the strains presented two groups (I and II) based on the expression of virulence genes related to the clinical diagnosis (Figure 1). Similarly, the cladogram of expression of the different gene members of the *ALS* and *SAP* families showed two groups (A and B), where group B was the most abundant, with the expression of 15 genes (*ALS* and *SAP*), whereas group A was represented only by the expression of three genes (*SAP3*, *SAP4*, and *ALS2*). Group I consisted of 54 strains (range of the no. of strains: 121–142) that were distributed into two subgroups, where the distribution of the expression of the *ALS* and *SAP* genes in the strains mainly associated with moderate periodontitis was higher than that in group II. In contrast, group II comprised 26 strains (range of the no. of strains: 140–14), distributed in several subgroups, and associated with the three diagnoses (gingivitis, moderate, and chronic periodontitis). Notably, several subgroups of group I had strains with the same expression profiles for the virulence genes *ALS* and *SAP*. In this subgroup, six strains (no. of strains: 213, 210, 187, 185, 55, and 153), all from moderate periodontitis, exhibited simultaneous expression of 17 of the 18 *ALS* and *SAP* genes analyzed. In addition, three other strains (no. of strains: 222, 212, and 219) from moderate periodontitis and gingivitis presented the same expression profile, consisting of 16 *ALS* and *SAP* genes. In one subgroup of group II, a pair of strains from patients with moderate periodontitis (no. of strains: 140 and 141) with the same profile of expressed virulence genes (12 *ALS* and *SAP* genes) was found. Notably, *ALS2* was the only gene not expressed in any strains studied (n = 80).

## 3. Discussion

Periodontitis is a chronic, multifactorial disease that affects 62% of adults [26] and is primarily caused by anaerobic bacteria [9]. The yeast *C. albicans* has also been described as a secondary pathogen associated with periodontitis [27]. In this study, *C. albicans* was more frequently isolated from patients with moderate periodontitis (66/80) than from gingivitis (6/80) or chronic periodontitis (8/80). Similar detection rates of *C. albicans* have been described: 20% in patients with periodontitis [5] and 25.6% in middle-aged and older patients with periodontitis [6].

Among the 268 patients studied, we also identified other bacteria commonly associated with periodontal disease, such as *Escherichia coli* (n = 50), *Klebsiella* spp. (n = 13), and *Staphylococcus aureus* (n = 50) [12,28,29]. These findings suggest that *C. albicans* may coexist with Gram-negative and Gram-positive bacteria to form polymicrobial biofilms, thereby intensifying local inflammation and contributing to the persistence of periodontal lesions. This hypothesis aligns with prior evidence of synergistic interactions between *C. albicans* and *Porphyromonas gingivalis* during co-infection [6].

Despite this growing body of evidence, the contribution of *C. albicans ALS* and *SAP* gene expression to periodontitis pathogenesis remains poorly characterized. Our group previously analyzed the distribution of gene association profiles of the lipase and phospholipase families related to antifungal resistance in the same periodontal strains of *C. albicans* [30]. Building on that work, the present study analyzed the different molecular expression patterns of *ALS* (*ALS1*, *ALS2*, *ALS3*, *ALS4*, *ALS5*, *ALS6*, *ALS7*, and *ALS9*) and *SAP* (*SAP1-SAP10*) family genes in *C. albicans* strains isolated from patients with periodontal disease in an in vitro infection model using an HGF cell line.

The presence of anaerobic red complex bacteria and the expression of virulence markers of *Porphyromonas gingivalis* (fimbriae, hemolysins, henaglutinin, and capsule), *Tannerella forsythia* (proteases, glycosidases, and outer membrane vesicles), and *Treponema denticola* (dentilisin, dentipain, main sheath protein, and motility and chemotaxis) [11] during periodontal disease may facilitate colonization by secondary pathogens associated with periodontitis, such as *C. albicans*. The collective expression of virulence markers of anaerobic red complex bacteria with the *ALS* and *SAP* genes of *C. albicans* may synergistically increase the pathogenicity of periodontal disease.

The *ALS* (agglutinin-like sequence) family genes of *C. albicans* encode large glycoproteins on the fungal cell surface and are involved in the adhesion process to host surfaces [31]. A wide distribution of *ALS* genes was detected globally in the strains across the three diagnoses, where *ALS1* (79/80), *ALS3* (70/80), *ALS4* (79/80), *ALS6* (77/80), *ALS7* (78/80), and *ALS9* (76/80) were found in most strains from gingivitis, moderate periodontitis, and chronic periodontitis. The elevated detection frequency of *ALS1* and *ALS3* genes in the strains associated with the three diagnoses may favor not only the adhesion and colonization of the gingival epithelium during periodontal disease but also the acuity and/or chronicity of the disease. This is supported by studies showing that *ALS1* and *ALS3* have been implicated in biofilm formation that promotes evasion of the host immune response and protection against the action of antifungals [32] and facilitates the formation of multispecies biofilms with other periodontal pathogens [33]. It has also been reported that the *ALS6*, *ALS7*, and *ALS9* genes, prevalent in most of the studied strains, can participate in biofilm formation, similar to the *ALS1* and *ALS3* genes, as demonstrated in an als3ΔΔ/als1ΔΔ3 mutant strain [34]. Furthermore, *C. albicans* can coaggregate with the commensal bacteria *S. gordonii*, *S. mitis*, *S. oralis*, and *S. sanguini*s as early colonizers, which, in turn, collaborate with secondary colonizers, such as *Fusobacterium nucleatum*, promoting coaggregation and additional adhesion of late colonizers responsible for the disease, such as *Porphyromonas gingivalis*, *Tannerella forsythia*, and *Treponema denticola* [33]. In this way, the presence of *C. albicans* could favor the formation of interspecies biofilms during periodontal infection.

Compared to our previous findings in *C. albicans* vaginal isolates, the periodontal strains showed higher detection frequencies of *ALS1*, *ALS2*, *ALS3*, *ALS4*, *ALS6*, *ALS7*, and *ALS9* in strains from the three diagnoses than those previously described by our working group in a study of *C. albicans* strains isolated from vaginal candidiasis [22]. In the in vitro infection model using the HGF cell line, the most frequently expressed genes in strains from gingivitis were *ALS1* (5/6), *ALS3* (5/6), *ALS4* (5/6), *ALS6* (6/6), and *ALS9* (6/6). On the other hand, in strains associated with moderate periodontitis, high percentages of expression were detected for *ALS1* (64/66), *ALS3* (58/66), *ALS4* (58/66), *ALS6* (64/66), *ALS7* (52/66), and *ALS9* (63/66). Among the strains isolated from patients with chronic periodontitis, the most frequently expressed genes were *ALS1* (8/8), *ALS6* (7/8), and *ALS7* (6/8).

We found high percentages of expression of *ALS* genes in periodontal strains of *C. albicans*, which are similar to the high percentages of expression of *ALS1–ALS7* genes described in a group of *C. albicans* strains (n = 37) isolated from patients with cystic fibrosis [35]. The high expression of the *ALS*1 and *ALS3* genes detected in our strains suggests their potential capacity to promote biofilm formation during periodontal disease. The *ALS1* and *ALS3* genes can be expressed in the hyphal form, which may favor tissue invasion and the severity of periodontal disease. High prevalence of expression of *ALS1*, *ALS2*, *ALS3*, and *ALS9* has also been described in strains from vaginal candidiasis using an infection model in a reconstituted human vaginal epithelium (RHVE) [36]. Furthermore, high percentages of expression for *ALS1*, *ALS2*, *ALS3*, *ALS4*, *ALS5*, and *ALS9* strains of *C. albicans* from oral candidiasis during the infection of a reconstituted human oral epithelium have been reported [24].

We also detected all 10 *SAP* genes (*SAP1–SAP10*) in gingivitis strains (6/6), while in moderate periodontitis, *SAP1*, *SAP6*, *SAP9*, and *SAP10* were detected in 100% (66/66), and in chronic periodontitis, *SAP1*, *SAP4*, *SAP6*, *SAP7*, *SAP8*, and *SAP10* were present in all strains. These high frequencies indicate that *SAP* genes are key virulence determinants involved in adhesion, tissue degradation, and immune evasion [37,38,39].

Although *SAP* gene expression has been widely studied in oral and vaginal candidiasis [40,41], *SAP* expression in *C. albicans* strains associated with periodontitis has not been extensively studied.

Using our HGF-based infection model, we observed high expression levels of *SAP1* (76/80), *SAP9* (78/80), and *SAP10* (77/80) across strains from all clinical groups. In contrast, *SAP2* (49/66), *SAP3* (38/66), *SAP4* (32/66), *SAP5* (41/66), *SAP6* (51/66), *SAP7* (46/66), and *SAP8* (44/66) were predominantly expressed in strains from moderate periodontitis, with lower frequencies in strains from gingivitis and chronic periodontitis. This expression pattern suggests that *SAP2*, *SAP3*, *SAP6*, and *SAP7* may contribute specifically to the acute phases of disease progression. For instance, Sap2 has proteolytic activity that degrades host proteins in the oral cavity [42], whereas Sap3 is mainly expressed in the yeast form and participates in the adhesion and colonization processes during the initial stage of infection [43]. Sap4–Sap6 favor the formation of hyphae [44] and, consequently, tissue invasion [45,46]. Sap7 could participate in hypha formation, Sap8 is associated with vaginal and oral infections [41], and Sap9 and Sap10 are involved in biofilm production in *C. albicans* strains [17]. The simultaneous expression of the 10 members of the *SAP* gene family in periodontal *C. albicans* strains was similar to that of an in vitro model of RHVE infection with *C. albicans* strains from vaginal candidiasis [47] and those in strains from oral candidiasis [48].

Interestingly, our reference strains of *C. albicans*, cv24, cv31, and cv42 from cervical and vaginal infections, have similar expression percentages of the *ALS* and *SAP* genes [22,47] to those found in this study, which leads us to assume that *C. albicans* strains from different types of candidiasis are capable of presenting distinct molecular arrangements of virolome expression.

The expression frequencies of the *ALS3*, *ALS4*, *ALS5*, *ALS9*, *SAP4*, *SAP5*, and *SAP*6 genes were lower in the strains isolated from chronic periodontitis compared to those from moderate periodontitis and gingivitis, which suggests that, in a more severe stage of the disease where there is a high destruction of tissues, these adhesion and protease genes are not essential.

Systematic, unsupervised hierarchical clustering revealed two major groups (I and II) based on gene expression profiles. Group I strains presented greater expression of the virulence genotypes (*ALS* and *SAP*) than group II strains. Notably, six strains from moderate periodontitis (213, 210, 187, 185, 55, 153) expressed 17 out of the 18 *ALS* and *SAP* genes analyzed. Three other strains (222, 212, 219) from moderate periodontitis (n = 2) and gingivitis (n = 1) expressed 16 genes. In contrast, two strains (140, 141) expressed only 12 genes.

Overall, 58 strains displayed distinct virulence gene expression profiles, highlighting the molecular diversity and adaptability of *C. albicans* in the periodontal environment. However, strains from different clinical diagnoses sharing identical profiles suggest that they may belong to the same *C. albicans* serotype. Therefore, whole-genome sequencing of the genomes of these strains in a subsequent study may provide more detailed information on the serotypes, the molecular composition of virulence, resistance to antifungals, and their relationship with the level of periodontal disease. Future studies should incorporate equivalent numbers of isolates from gingivitis, moderate periodontitis, and chronic periodontitis to improve the robustness of the virulome expression signatures. We acknowledge the uneven distribution of participants across diagnostic groups, which is determined by two main factors. This is a descriptive, observational study based on individuals who attended our dental clinic, where the prevalence of gingivitis cases is inherently lower than that of periodontitis. While this may create the appearance of a sampling imbalance, it reflects the natural distribution of both the condition and the pathogen within the studied population. These efforts will contribute to better vaccine design and optimization of antifungal treatment strategies for *C. albicans*-associated periodontal disease.

## 4. Materials and Methods

### 4.1. Patient Selection

This study was conducted at the Endoperiodontology Clinic of the Iztacala Faculty of Advanced Studies, National Autonomous University of Mexico (UNAM). A group of patients with periodontal disease (n = 268) and a control group of healthy individuals without any clinical signs of periodontal disease (n = 100) were included. All the patients enrolled in this study signed an informed consent form. This study was approved by the Ethics and Biosafety Committee of the Iztacala School of Advanced Studies, UNAM (approval No. 00012). Patients who were smokers, who were undergoing periodontal cleaning, or who had received antibiotic treatment in the previous six months were excluded from this study [49]. Patients with cancer treatment or treatment for any other progressive immune disease were excluded from this study. The three diagnoses of periodontal disease were established by endoperiodontology specialists (FES Iztacala, UNAM) using a Williams Fox periodontal probe (Hu-Friedy Mfg. Co., LLC, Chicago, IL, USA). The diagnostic criteria for periodontitis were loss of periodontal tissue attachment, periodontal pocket formation, alveolar bone loss, gingival recession, and tooth mobility. Patients with moderate periodontal disease presented with a tooth attachment loss of 2–4 mm, and those with chronic periodontal disease presented with a tooth attachment loss greater than or equal to 5 mm [49]. Patients with gingivitis presented exclusively with irritation, redness, and tissue inflammation; sensitivity; bleeding when brushing their teeth; and gums that were soft to the touch. Samples were collected from the periodontal pockets of the incisors, canines, premolars, or molars of patients with moderate or chronic periodontitis using sterile paper points. Patients with gingivitis did not present with periodontal pockets, so samples from the gingival surfaces were taken with sterile swabs. The samples were placed in brain–heart infusion (BHI; MCD LAB, Tlalnepantla, Mexico State) nutrient broth and transported to the Clinical Laboratory of CUSI (Clínica Universitaria de la Salud Integral), FES Iztacala, UNAM, for microbiological culture. The BHI cultures were incubated at 37 °C for 24 h. They were then plated on Sabouraud agar (MCD LAB, Tlalnepantla, Mexico City), eosin methylene blue (EMB; MCD LAB, Tlalnepantla, Edo. Mexico), and S110 (MCD LAB, Tlalnepantla, Edo. de México) and incubated at 37 °C for 24 h. The *K. pneumoniae* was identified by PCR based on the *16S–23S rDNA* internal transcribed spacer [50], *E. coli* strains were identified by standard IMViC biochemical tests (Indole, Methyl Red, Voges-Proskauer and Citrate; MCD LAB, Tlalnepantla, Edo. Mexico), and *S. aureus* strains were identified by bacteriological and biochemical tests that included mannitol, coagulase, and polymerase chain reaction (PCR) by amplification of the *23S rRNA*, *nuc*, *spax* region, and *coa* genes [51].

### 4.2. DNA Extraction

After incubation in BHI (MCD LAB, Tlalnepantla, Mexico State) at 37 °C for 24 h for samples taken from periodontal pockets of patients with moderate periodontitis and chronic periodontitis and from gums of patients with gingivitis and healthy control individuals, the samples were streaked by the cross-streak method using sterile loops on Sabouraud agar at 37 °C for 24 h. The *Candida albicans* species of pure cultures on Sabouraud agar were identified by the germ tube test in BHI (MCD LAB, Tlalnepantla, Mexico State) broth containing 10% horse serum and confirmed by the polymerase chain reaction (PCR) method, for which DNA extraction from the strains was performed by the boiling method as previously described [52]. From the fungal culture on Sabouraud agar, six colonies of approximately 2 mm in diameter of each strain were picked using a sterile loop and placed in Eppendorf tubes containing 1.5 mL of sterile deionized water. The tubes were boiled for 20 min and subsequently incubated on ice for 10 min. Finally, they were centrifuged at 10,000 rpm for 10 min, and the supernatant containing the DNA was separated and stored in another Eppendorf tube at −20 °C until used for PCR.

### 4.3. Identification of C. albicans

Using the DNA obtained from each strain by the boiling method, *C. albicans* was identified by PCR through amplification of internal transcribed spacers (ITS) 1 and 2 of the *rRNA* gene (Table 5), as previously described [53]. For each singleplex PCR assay, a final volume of 25 μL was used per reaction mixture: 12.5 μL of Taq DNA Polymerase Master Mix RED (Ampliqon, Copenhagen, Denmark), 1 μL of forward primer, 1 μL of reverse primer (10 pmol, Integrated DNA Technologies, San Diego, CA, USA: Table 5), 3 μL of DNA template (100 ng), and 7.5 μL of nuclease-free water. The PCR amplification conditions were as follows: initial denaturation at 96 °C for 5 min, followed by 40 cycles of 94 °C for 30 s, 58 °C for 30 s, and 72 °C for 30 s. Finally, the final extension was at 72 °C for 15 min. The strain used as a positive control was *C. albicans* ATCC32354. All PCR assays were performed in triplicate. The amplicons were stained with Midori Green (Nippon Genetics, Düren, Germany, EUROPE) after electrophoresis in 2% agarose gels, which were photographed under UV illumination with GEL LOGIC 100 (Kodak, Carestream Molecular Imaging, Rochester, NY, USA) equipment.

### 4.4. Identification of ALS Genes in Strains

*ALS* family genes were detected using uniplex PCR, as previously described [24] (Table 5). The final volume of the reaction mixture was 25 μL: 12.5 μL of Taq DNA Polymerase Master Mix RED (Ampliqon, Copenhagen, Denmark), 1 μL of forward primer, 1 μL of reverse primer (10 pmol, Integrated DNA Technologies, San Diego, CA, USA; Table 5), 3 μL of DNA template (100 ng), and 7.5 μL of nuclease-free water. DNA amplification was performed under the following conditions: initial denaturation at 94 °C for 5 min, followed by 40 cycles at 94 °C for 30 s, 58 °C for 30 s, and 72 °C for 30 s. Finally, an extension was performed at 72 °C for 7 min. *C. albicans* strains cv24, cv31, and cv42 from cervicovaginal infections belonging to the strain library of the CUSI Clinical Laboratory [22,47], FES Iztacala, and strain *C. albicans* ATCC32354 were used as positive controls. All PCR assays were performed in triplicate.

### 4.5. Identification of SAP Genes in Strains

*SAP* family genes were detected using uniplex PCR, as previously described [47] (Table 5). The final volume of the reaction mixture was 25 μL: 12.5 μL of Taq DNA Polymerase Master Mix RED (Ampliqon, Copenhagen, Denmark), 1 μL of forward primer, 1 μL of reverse primer (10 pmol, Integrated DNA Technologies, San Diego, CA, USA: Table 5), 3 μL of DNA template (100 ng), and 7.5 μL of nuclease-free water. The amplification conditions of the *SAP* genes were as follows: initial denaturation at 95 °C for 15 min, followed by 45 cycles at 95 °C for 15 s, 58 °C for 30 s, and 60 °C for 60 s. Finally, an extension was performed at 72 °C for 5 min. *C. albicans* strains cv24, cv31, and cv42 from cervical and vaginal infections as well as strain *C. albicans* ATCC32354 were used as positive controls. All PCR assays were performed in triplicate.

### 4.6. Preparation of Gingival Fibroblasts

The HGF cell line (ATCC, CRL-2104) was seeded at a density of 5 × 10^3^ cells per cm^2^ and cultured in 75 cm^2^ Corning cell culture flasks in a water-saturated atmosphere at 37 °C and 5% CO_2_. Fibroblasts were maintained in Dulbecco’s modified high glucose Eagle’s medium (Sigma Aldrich, Saint Louis, MO, USA), supplemented with 10% fetal bovine serum (previously inactivated at 56 °C for 30 min; GIBCO BRL, Gaithersburg, MD, USA), containing 10 U of penicillin/25 μg of streptomycin/mL (Sigma Aldrich). Fibroblasts were cultured to confluence at a density of 2.5 × 10^5^ cells/mL, washed twice with phosphate-buffered saline, and dissociated with 0.25% trypsin and 1 mM EDTA for 5 min at 37 °C and 5% CO_2_. The cells were subsequently collected and placed in triplicate into the plate wells. Finally, the plate was incubated in the incubator at 37 °C with 5% CO_2_ for 48 h until a confluence of 1.8 × 10^5^ cells/well was obtained.

### 4.7. Dilution of C. albicans Yeasts

One colony of each *C. albicans* strain was inoculated in triplicate into 2 mL of brain–heart infusion broth (MCD Lab, Edo. de México, Mexico) and incubated at 37 °C for 12 h with constant shaking. Dilutions of each culture were prepared at a 1:5 ratio using phosphate-buffered saline to obtain an optical density of 0.125 at 600 nm (OD600) using a Beckman DU-7400 spectrophotometer (Laguna Hills, CA, USA), equivalent to a concentration of 2 × 10^6^ cells/mL.

### 4.8. In Vitro Infection of Fibroblasts

To simulate periodontal infection, we used a previously described in vitro infection model [24,54]. Fifty microliters at a concentration of 2 × 10^6^ cells/mL of each *C. albicans* strain culture was added to a monolayer (1.8 × 10^5^ cells/well) of HGFs (ATCC, CRL-2104). Triplicate 24-well plates were incubated with 1 mL of F12K (Sigma Aldrich; Merck KGaA, Darmstadt, Germany) and 10% fetal bovine serum at 37 °C for 48 h in a 5% CO_2_ atmosphere with saturated humidity. *C. albicans* strains cv24, cv31, and cv42 and *C. albicans* ATCC32354 were used as controls, and yeast adherence to fibroblasts was verified under a light microscope to ensure fungal viability.

### 4.9. C. albicans RNA Extraction and Reverse Transcription to cDNA

Triplicate *C. albicans* strains were harvested from the surface of the HGF cell line (ATCC, CRL-2104), resuspended in 1000 μL of RNA Protect Bacteria reagent (Qiagen, Hilden, Germany), and centrifuged at 8000 rpm for 5 min to obtain fungal cell pellets. *C. albicans* RNA was extracted using the QIAcube automated workstation (Qiagen, Hilden, Germany) with the RNeasy Mini commercial kit (Qiagen), which included a 200 μL treatment with buffer Y1 (Qiagen, Hilden, Germany) containing lyticase (50 U/107 cells) to disrupt the fungal cell wall. The concentration and purity of total RNA were measured using a NanoDrop 2000 spectrophotometer (Thermo Fisher Scientific, Waltham, MA, USA). A QuantiTect Reverse Transcription Kit (Qiagen, Hilden, Germany) was used for first-strand cDNA synthesis.

### 4.10. Determination of Virulence Gene Expression in Strains by Real-Time PCR

To determine the expression of *ALS* and *SAP* family genes in *C. albicans* strains, we used the Corbette^®^ Real-Time PCR device (Rotor-Gene Q 5plex; Qiagen, Hilden, Germany). The final volume per reaction mixture was 25 µL: 12.5 µL of the SYBR Green Master Mix, QuantiNova SYBR Green PCR (Qiagen, Hilden, Germany), 1 µL of each forward and reverse oligonucleotide (10 pmol, Integrated DNA Technologies, San Diego, CA, USA; Table 5), 1 µL of *cDNA* (100 ng/µL), and 9.5 µL of nuclease-free H2O. The real-time PCR conditions were as follows: one HotStart activation cycle at 95 °C for 2 min, 40 denaturation cycles at 95 °C for 5 s, and a 60 °C annealing/extension combination for 10 s. For each quantitative real-time PCR assay, a standard curve was constructed from three *cDNA* dilutions (100, 200, and 300 ng/µL) of a strain containing the gene of interest (positive control). Rotor-Gene Q 5plex HRM System software version 2.3.1.49 (Qiagen) was used to calculate the threshold cycle (CT) limit for each strain. Thus, using the CT values of each strain obtained with respect to the CT of the control strain standard curve, the arithmetic mean was obtained, and the percentage of expression of each gene was determined. For each real-time PCR run, the melting point (dissociation curve of specific products) and *ACT1* and *RPP2B* genes were included as housekeeping genes. *C. albicans* strains cv24, cv31, and cv42 from cervical and vaginal infections [22] and strain ATCC32354 were used as positive controls, and a negative control (no temperature control) was also used. All real-time PCR assays were performed in triplicate.

### 4.11. Statistical Analysis

The chi-square test was applied using SPSS statistical software (version 20.0; SPSS Inc., Chicago, IL, USA) (*p* < 0.05) to establish the association between the frequency of the eight adhesion genes of the *ALS* family and ten proteinase genes of the *SAP* family of the strains related to the three diagnoses (gingivitis, moderate periodontitis, and chronic periodontitis) as well as the association between the frequency of expression of the *ALS* and *SAP* genes in the *C. albicans* strains related to the three diagnoses (gingivitis, moderate periodontitis, and chronic periodontitis).

### 4.12. Unsupervised Hierarchical Clustering

To investigate the relationship between *C. albicans* gene expression and periodontal stage, unsupervised hierarchical clustering was performed using Gower’s similarity coefficient [55]. The analysis was based on a categorical data matrix constructed in R (v3.6.1) with the cluster package (v2.1.0), which integrated RT-PCR detected presence or absence of *ALS* and *SAP* virulence genes together with the clinical stage of periodontal disease (gingivitis, moderate periodontitis, chronic periodontitis). The distance between each strain was calculated based on the overall similarity coefficient, which was used to estimate the maximum possible absolute discrepancy between each matched pair of strains. After calculating the distances, we clustered mutually exclusive groups using Ward’s method in R [56]. The strains were visualized using a genotype expression distribution diagram, and a dendrogram was constructed using Complex heatmap (v3.6.2, R core).

## 5. Conclusions

The findings of this study highlight the widespread expression of the *C. albicans* virolome as a secondary pathogen associated with periodontal disease. The molecular properties involved in adhesion, biofilm formation, tissue degradation, and host immune evasion detected in *C. albicans* strains could contribute to the severity of periodontitis. Therefore, screening and medical treatment regimens are key against this opportunistic pathogen in patients with periodontal disease.

## Figures and Tables

**Table 1:** Distribution of *ALS* genes in *C. albicans* strains associated with periodontal disease.

Function	Gene	Strain Origin (n = 80)			*p*-Value	Total (n = 80)
		Gingivitis (n = 6) No. (%)	Moderate Periodontitis (n = 66) No. (%)	Chronic Periodontitis (n = 8) No. (%)		
	*ALS1*	6 (100)	65 (98.5)	8 (100)	1	79
Adhesion	*ALS2*	5 (83.3)	45 (68.1)	5 (62.5)	0.7189	55
	*ALS3*	5 (83.3)	58 (87.8)	7 (87.5)	0.8202	70
	*ALS4*	6 (100)	65 (98.4)	8 (100)	1	79
	*ALS5*	4 (66.6)	48 (72.7)	4 (50)	0.3571	56
	*ALS6*	6 (100)	64 (96.9)	7 (87.5)	0.443	77
	*ALS7*	6 (100)	64 (96.9)	8 (100)	1	78
	*ALS9*	6 (100)	63 (95.4)	7 (87.5)	0.5443	76

**Table 2:** Distribution of *SAP* genes in *C. albicans* strains associated with periodontal disease.

Function	Gene	Strain Origin (n = 80)			*p*-Value	Total (n = 80)
		Gingivitis (n = 6) No. (%)	Moderate Periodontitis (n = 66) No. (%)	Chronic Periodontitis (n = 8) No. (%)		
Secreted aspartyl protease	*SAP1*	6 (100)	66 (100)	8 (100)	1	80
	*SAP2*	6 (100)	65 (98.4)	7 (87.5)	0.3212	78
	*SAP3*	6 (100)	62 (93.9)	7 (87.5)	0.6282	75
	*SAP4*	6 (100)	64 (96.9)	8 (100)	1	78
	*SAP5*	6 (100)	55 (83.3)	5 (62.5)	0.1509	66
	*SAP6*	6 (100)	66 (100)	8 (100)	1	80
	*SAP7*	6 (100)	65 (98.4)	8 (100)	1	79
	*SAP8*	6 (100)	63 (95.4)	8 (100)	1	77
	*SAP9*	6 (100)	66 (100)	8 (100)	1	80
	*SAP10*	6 (100)	66 (100)	8 (100)	1	80

**Table 3:** Analysis of *mRNA* expression of *ALS* genes in strains associated with periodontal disease. Note: Significant *p*-values (<0.05) are shown in bold.

Function	Gene	Strain Origin (n = 80)			*p*-Value	Total (n = 80)
		Gingivitis (n = 6) No. (%)	Moderate Periodontitis (n = 66) No. (%)	Chronic Periodontitis (n = 8) No. (%)		
	*ALS1*	5 (83.3)	64 (98.5)	8 (100)	0.2342	77
Adhesion	*ALS2*	0 (0)	0 (0)	0 (0)	**-**	0
	*ALS3*	5 (83.3)	58 (87.8)	4 (50)	**0.02123**	67
	*ALS4*	5 (83.3)	58 (87.8)	4 (50)	**0.02123**	67
	*ALS5*	2 (33.3)	44 (66.6)	4 (50)	0.1845	50
	*ALS6*	6 (100)	64 (96.9)	7 (87.5)	0.443	77
	*ALS7*	4 (66.6)	52 (78.7)	6 (75)	0.7575	62
	*ALS9*	6 (100)	63 (95.4)	4 (50)	**0.00264**	73

**Table 4:** Analysis of *mRNA* expression of *SAP* genes in strains associated with periodontal disease. Note: Significant *p*-values (<0.05) are shown in bold.

Function	Gene	Strain origin (n = 80)			*p*-Value	Total (n = 80)
		Gingivitis (n = 6) No. (%)	Moderate Periodontitis (n = 66) No. (%)	Chronic Periodontitis (n = 8) No. (%)		
Secreted aspartyl protease	*SAP1*	6 (100)	62 (93.9)	8 (100)	1	76
	*SAP2*	2 (33.3)	49 (74.2)	3 (37.5)	**0.01577**	54
	*SAP3*	1 (16.6)	38 (57.5)	2 (25)	**0.04985**	41
	*SAP4*	2 (33.3)	32 (48.4)	3 (37.5)	0.6255	37
	*SAP5*	3 (50)	41 (62.1)	3 (37.5)	0.3722	47
	*SAP6*	3 (50)	51 (77.2)	3 (37.5)	**0.02604**	57
	*SAP7*	1 (16.6)	46 (69.7)	3 (37.5)	**0.008886**	50
	*SAP8*	3 (50)	44 (66.6)	5 (62.5)	0.7372	52
	*SAP9*	5 (83.3)	65 (98.4)	8 (100)	0.1541	78
	*SAP10*	6 (100)	63 (95.4)	8 (100)	1	77

**Table 5:** Oligonucleotides used for the identification of *C. albicans* and virulence genes.

Gene	Sequence (5′-3′)	Amplicon Size (bp)
ITS1 ITS2	TTTATCAACTTGTCACACCAGA	273
	ATCCCGCCTTACCACTACCG	
*ALS1*	GACTAGTGAACCAACAAATACCAGA	318
	CCAGAAGAAACAGCAGGTGA	
*ALS2*	CCAAGTATTAACAAAGTTTCAATCACTTAT	366
	TCTCAATCTTAAATTGAACGGCTTAC	
*ALS3*	CCACTTCACAATCCCCATC	342
	CAGCAGTAGTAGTAACAGTAGTAGTTTCATC	
*ALS4*	CCCAGTCTTTCACAAGCAGTAAAT	356
	GTAAATGAGTCATCAACAGAAGCC	
*ALS5*	TGACTACTTCCAGATTTATGCCGAG	318
	ATTGATACTGGTTATTATCTGAGGGAGAAA	
*ALS6*	GACTCCACAATCATCTAGTAGCTTGGTTT	152
	CAATTGTCACATCATCTTTTGTTGC	
*ALS7*	GAAGAGAACTAGCGTTTGGTCTAGTTGT	206
	TGG CATACTCCAATCATTTATTTCA	
*ALS9*	CCATATTCAGAAACAAAGGGTTC	198
	AACTGAAACTGCTGGATTTGG	
*SAP1*	TCAATCAATTTACTCTTCCATTTCTAACA	161
	CCAGTAGCATTAACAGGAGTTTTAATGACA	
*SAP2*	AACAACAACCCACTAGACATCACC	178
	TGACCATTAGTAACTGGGAATGCTTTAGGA	
*SAP3*	CCTTCTCTAAAATTATGGATTGGAAC	231
	TTGATTTCACCTTGGGGACCAGTAACATTT	
*SAP4*	TTATTTTTAGATATTGAGCCCACAGAAA	171
	GCCAGTGTCAACAATAACGCTAAGTT	
*SAP5*	AGAATTTCCCGTCGATGAGACTGG	277
	CAAATTTTGGGAAGTGCGGGAAGA	
*SAP6*	CCCGTTTTGAAATTAAATATGCTGATGG	187
	GTCGTAAGGAGTTCTGGTAGCTTCG	
*SAP7*	GAAATGCAAAGAGTATTAGAGTTATTAC	196
	GAATGATTTGGTTTACATCATCTTCAACTG	
*SAP8*	TCTCAAGAAATTATCCCCCAAAATA	256
	TCGGTTCCATTATCAGAATTTGTTC	
*SAP9*	ATTTACTCCACAGTTTATATCACTGAAGGT	80
	CCACCAGAACCACCCTCAGTT	
*SAP10*	CCCGGTATCCAATAGAATCGAA	80
	TCAGTGAATGTGACGAATTTGAAGA	

## Data Availability

The original contributions presented in this study are included in the article.
